# Choosing the negative: A behavioral demonstration of morbid curiosity

**DOI:** 10.1371/journal.pone.0178399

**Published:** 2017-07-06

**Authors:** Suzanne Oosterwijk

**Affiliations:** 1 Department of Social Psychology, University of Amsterdam, Amsterdam, The Netherlands; 2 Amsterdam Brain and Cognition Centre, Amsterdam, The Netherlands; University of Würzburg, GERMANY

## Abstract

This paper examined, with a behavioral paradigm, to what extent people choose to view stimuli that portray death, violence or harm. Based on briefly presented visual cues, participants made choices between highly arousing, negative images and positive or negative alternatives. The negative images displayed social scenes that involved death, violence or harm (e.g., war scene), or decontextualized, close-ups of physical harm (e.g., mutilated face) or natural threat (e.g., attacking shark). The results demonstrated that social negative images were chosen significantly more often than other negative categories. Furthermore, participants preferred social negative images over neutral images. Physical harm images and natural threat images were not preferred over neutral images, but were chosen in about thirty-five percent of the trials. These results were replicated across three different studies, including a study that presented verbal descriptions of images as pre-choice cues. Together, these results show that people deliberately subject themselves to negative images. With this, the present paper demonstrates a dynamic relationship between negative information and behavior and advances new insights into the phenomenon of morbid curiosity.

## Introduction

People are curious of highly intense negative information. This phenomenon, often referred to as morbid curiosity [1], can be inferred from the popularity of horror movies and crime shows; the observation that people seek out coverage of violence in the news and on the internet; and the existence of phenomena such as “disaster-tourism” and “rubbernecking”. In this paper, the term morbid curiosity is used to specify curiosity for information involving death, violence or harm, but not an “unhealthy” or “abnormal” form of curiosity.

Even though many people know morbid curiosity from experience, this important psychological phenomenon has received little attention in psychological science to date [2–5]. Most studies presenting highly intense, negative stimuli focus on avoidance states such as fear and disgust (e.g., [6, 7]) or on basic dimensions of affective experience such as arousal and valence (e.g., [8, 9]). As a result, the current understanding of how people respond to negative stimuli is incomplete, and reflects a gap between the scientific study of affective phenomena, and the observation that, in daily life, people often approach and explore negative stimuli. In order to generate more insight into the dynamic way in which people respond to negative information, the present paper aims to map the phenomenon of morbid curiosity by asking a simple question: if you give people a choice, do they actually choose to view images that portray death, violence or harm?

### Curiosity and interest for negative information

Although the topic of morbid curiosity has not received much attention, several authors have proposed insightful definitions and explanations of general curiosity. For example, Litman [10] defines curiosity as ‘a desire to know, to see or to experience, that motivates exploratory behavior directed towards the acquisition of new information’ (p.793). According to Loewenstein [11] people experience curiosity when there is an “information gap” between what they want to know and what they currently know. Curiosity is reflected in seeking out information that can reduce or resolve this discrepancy (see also [12]). Although these views leave the valence of the information that is sought unspecified, Silvia [13] has emphasized that both positive *and* negative stimuli can give rise to interest, a concept closely related to curiosity [14].

Although most studies investigating curiosity have focused on curiosity for positive stimuli or knowledge-oriented stimuli, such as trivia (e.g., [12, 15–17] see for an overview [11]), there are some studies that specifically targeted whether negative stimuli give rise to interest or curiosity. For example, Zuckerman and Litle [1] demonstrated a positive relationship between the personality trait of sensation seeking (i.e., the need to subject yourself to arousing and novel experiences) and self-reported curiosity for morbid events (e.g., enjoyment in watching violence or death in films, sport or news). A more recent example is the work of Turner and Silvia [18] who presented participants with images of calming art and disturbing art and asked participants for ratings of interest and pleasantness. Their findings demonstrated that there was no relationship between interest and pleasantness; people found disturbing art both unpleasant *and* interesting. Rimé and colleagues [3] focused on the phenomenon of “morbid fascination” and collected reports of attention and interest for pictures of the terror attacks of 9/11. According to their results, about half of the participants were characterized by their interest for the negative scenes. Some of these participants experienced an ambivalent mix of interest and specific emotional reactions (e.g., disgust or anxiety) whereas other participants experienced “cold” fascination for the negative scenes that was solely defined by interest and high attention.

Even though some work has been done on subjectively reported curiosity or interest for negative information, relatively little is known about the behavioral aspects of this phenomenon. Recently, Hsee and Ruan [19] performed a study that explicitly targeted people’s decisions to expose themselves to aversive stimuli. They found that participants showed more curiosity for aversive stimuli with an uncertain outcome, as compared to aversive stimuli or neutral stimuli with a certain outcome. This effect was found for decisions to expose oneself to electric shocks, unpleasant sounds and images of insects. Although these studies provide important new insights regarding the role of uncertainty in curiosity for negativity, this work does not allow for conclusions about choice for “morbid” stimuli that portray violence, death or harm. The present paper reports several studies that explicitly target people’s choice for stimuli with such content.

### A focus on behavior

In daily life people commonly *seek out* negative information. The studies reported in the present paper aim to mimic this behavioral expression by implementing a paradigm that provides participants with a choice to approach or avoid stimuli that portray death, violence or harm. This operationalization connects to theoretical views that define curiosity in terms of explorative or information-seeking behavior [10, 14, 20].

The focus on behavior is important, because so far, most studies that explicitly targeted interest or curiosity for negative information examined subjective ratings (e.g., [3, 18]; see for exceptions [19, 21]) or studied the relationship between self-reported behavior and personality measures [1, 22, 23]. However, self-report and behavior do not always align [24], and it is therefore important to utilize a behavioral paradigm when testing the prevalence of curiosity for negative information. Other relevant work has focused on subjective reports of *enjoyment* of negative or violent information (e.g., [25–27]). Nevertheless, it has been suggested in the literature that morbid curiosity is a phenomenon characterized by “wanting” (i.e., incentive salience or approach motivation) as opposed to “liking” [10], and therefore a behavioral paradigm seems better suited to study this phenomenon.

Furthermore, it is important to distinguish curiosity from mere attention [11]. Although previous work has provided some insight into morbid curiosity by demonstrating increased viewing time for negative images over neutral images (e.g., [28–30]), viewing time does not *necessarily* indicate interest or curiosity, but may also reflect a state of general attentional vigilance or a state of anxiety. For example, individuals with generalized anxiety disorder or phobic individuals find it difficult to disengage their attention from stimuli relevant to their disorder, such as angry faces or pictures of spiders (see for an overview [31, 32]). Thus, enhanced attention towards negative stimuli may reflect curiosity or interest, but it can also indicate other affective processes.

Finally, most work reviewed above involved research methods in which participants were *confronted* with negative stimuli. Nevertheless, in the context of morbid curiosity it is important to emphasize that people are active agents. People choose, voluntarily, to view images on a website, watch documentaries, or read articles with morbid content. Therefore, the present studies do not measure how people respond to a morbid stimulus once it is there, but rather, as a more direct indicator of morbid curiosity, whether people voluntarily expose themselves to stimuli that portray violence, death or harm.

### The present studies

The present paper has two aims. The first aim is to examine, in a carefully controlled experimental setting, whether people deliberately choose to view images that portray death, violence or harm when they have a non-negative alternative option that would allow them to avoid the negative images. The main hypothesis tested with regard to this aim follows from theoretical accounts that emphasize that curiosity is characterized by exploratory behavior [10, 14, 20]. Although the existence of morbid curiosity could be argued when some exploration of images portraying death, violence or harm occurs, a strong hypothesis regarding morbid curiosity would state that people *prefer* to explore images with such content over non-negative alternatives. This hypothesis was tested by asking participants to choose between viewing a negative image and a neutral alternative (Study 1, 2 and 3) and between viewing a negative image and a positive alternative (Study 2 and 3). To evoke an information gap [11] participants were either cued to make their choice by two briefly presented images in small format (Study 1 and 2) or by two verbal descriptions of the negative and alternative image (Study 3). When the choice was made, participants were presented with the chosen image in full screen format.

In order to advance the understanding of morbid curiosity, it is important to engage in hypothesis-generating research. Therefore, the second aim of the present paper is to explore whether different *types* of negative information affect curiosity in a similar (or dissimilar) way. For this purpose, the choice paradigm included three categories that reflected different portrayals of death, violence or harm in terms of contextual information, social interaction and the presence of human actors. The negative *social* category involved images of death, violence or harm in a social context, such as interacting victims and/or perpetrators of violence (e.g., a man beaten up in a subway), or people responding to or observing dead or wounded people (e.g., a group of people standing around a dead body). These images always displayed multiple people and emphasized interpersonal interaction. The negative *physical* category involved stimuli that displayed close-up, graphic portrayals of death or physical harm, such as mutilations (e.g., a severed hand) or single dead bodies (e.g., a body with a slit throat in a morgue). These images always displayed a single person or body part and did not involve interpersonal interaction. The negative nature category (only included in Study 1 and 2) involved images that displayed close-up, decontextualized images of attacking animals (e.g., attacking shark) or passive insects (e.g., a group of cockroaches). No humans were present in the negative nature images.

Although there was no theoretical basis for distinguishing between these three categories, there were two practical observations that lead to examining choice behavior for social, physical and nature images separately. The first observation was that these three categories connect to particular types of negative content that people can encounter in daily life; namely negative content with a focus on a social narrative (e.g., crime shows/documentaries, thrillers); negative content with a focus on graphic harm or gore (e.g., horror movies, online videos of harm); and negative content that involves “dangerous” animals (e.g., shark videos/ documentaries). The second observation was that stimuli from these three categories are used in affective science to target different discrete emotions [7, 33]. For example, images from the negative physical category have been used in research that specifically targeted disgust [6, 30, 34], whereas images from the negative nature category have been used in research that specifically targeted fear or threat [35, 36]. Images that display humans in negative social situations are associated in the literature with a variety of discrete negative emotions, such as sadness, disgust and fear [7] and have been used in studies that specifically target empathy [37, 38]. Considering these two observations, it was decided not to collapse across negative stimuli, but instead to measure choice behavior for social, physical and nature images separately.

Since the second aim of this paper is exploratory in nature, no specific hypotheses regarding curiosity for the three categories were formulated at the start of the project. These hypotheses were based on the findings of a pilot study and subsequently pre-registered and tested in confirmatory fashion in Study 1. This procedure is consistent with current guidelines in the literature for exploratory or hypothesis-generating research [39, 40].

## Study 1

The first step in the present series of studies was to investigate choice for negative stimuli with a pilot study. This pilot study tested the hypothesis that participants would prefer to view negative images over neutral images, and explored whether this preference held for each of the negative image categories (i.e., social, physical and nature). For the sake of brevity, all details concerning this pilot study are placed in S1 Pilot Study; the mean choice proportions are presented in Table 1. Following the pilot study, two central hypotheses were tested in confirmatory fashion in Study 1. Firstly, Study 1 tested the hypothesis that participants would prefer to view social negative images over neutral images. Secondly, Study 1 tested the hypothesis that the proportion chosen negative social images would be significantly higher than the proportion chosen negative physical and negative nature images.

**Table 1:** Overview of choice proportions across studies.

	Pilot	Study 1	Study 2	Study 3
Neg social–neutral	.70*^a^	.62*^a^	na.	na.
Neg physical–neutral	.53^b^	.43^b^	na.	na.
Neg nature–neutral	.47^b^	.35*^b^	na.	na.
Neg social–neg physical	.66*	.64*	na.	na.
Neg social–neg nature	.64*	.66*	na.	na.
Neg physical–neg nature	.46	.48	na.	na.
Neg social–neu social	na.	na.	.58†^d^	.67*^c^
Neg physical–neu physical	na.	na.	.36*^abc^	.35*^a^
Neg nature–neu nature	na.	na.	.39*^ac^	na.
Neg social–pos social	na.	na.	.43†^c^	.47^b^
Neg physical–pos physical	na.	na.	.35*^ab^	.32*^a^
Neg nature–pos nature	na.	na.	.30*^b^	na.

* comparison against .5; *p* < .05, corrected

^†^ comparison against .5; *p* < .05, uncorrected.

Note. Numbers with different alphabetical superscripts differ significantly from each other (*p* < .05, corrected). For transparency, the results from the pilot study and the choice proportions for the negative—negative choice conditions are also included.

Study 1 further incorporated subjective ratings of interest, negativity, intensity and complexity. Subjective ratings of interest were collected because curiosity and interest are seen as closely related concepts, or synonyms [13, 14]. Because of the theoretical relationship between these concepts, it is expected that the measures of these concepts will show a positive relationship. More specifically, it is predicted that the proportion chosen negative images of a particular category (as an indicator of curiosity) should correlate positively with the mean interest ratings for the images of that category. The convergence between these two measures would support the notion that the choices that participants make in the choice paradigm are a reflection of curiosity.

Subjective ratings of negativity and intensity were collected to explore possible relationships between these dimensions and choice behavior. A positive relationship between the rated intensity of negative images and choice proportion may be predicted based on a sensation seeking account that poses that curiosity for negative information is motivated by a need to seek out arousing experiences [1]. A negative relationship between the rated negativity of negative images and choice proportion may be predicted based on the theoretical assumption that negative information is inherently associated with avoidance behavior [41]. Finally, subjective ratings of complexity were collected because some authors have argued for a relationship between the complexity of stimuli and perceivers’ interest or curiosity [13, 20].

### Method

#### Participants

Even though the exploratory pilot study demonstrated large effect sizes (*d’s* > .60), a conservative estimate of effect size was used to determine the minimum sample size for Study 1. A calculation performed in G*Power [42], with *d* = .40, α = .05 and 1-β = .80 as parameters, determined a minimum sample size of 52 participants.

Fifty-three students (39 females; Mean age = 22.7; SD = 5.76) from the University of Amsterdam signed the informed consent form. Participants were warned in the information brochure that the study consisted of material that could be experienced as shocking. The study was approved by the Ethics Review Board of the Psychology Department at the University of Amsterdam (2014-SP-3920). Students participated for course credit or financial compensation. Prior to analysis, three participants were excluded from the sample. One participant was excluded because of missing data; one participant was excluded because the experimenter severely doubted the motivation of the participant; and one participant stopped participation because he/she felt uncomfortable.

#### Design

The factor *category* (social vs. physical vs. nature) reflected whether the negative stimulus had social, physical harm or nature content. The factor *combination* (neutral vs. negative) reflected whether the negative stimulus was paired with another negative or with a neutral alternative. Both factors were varied within participants. The design, stimuli, hypotheses and analysis protocol for Study 1 were pre-registered on the Open Science Framework [43].

#### Materials

Four categories of images were selected from the International Affective Picture System database (IAPS; [44]) and the image set developed by the Kveraga lab (http://www.kveragalab.org/stimuli.html; [45]). The negative social category consisted of thirty images displaying social situations involving death, violence or harm, such as war scenes, accidents, conflicts or attacks. Negative social images always involved multiple people. The negative physical category consisted of thirty images displaying decontextualized close-ups of severe bodily harm, such as mutilated hands, bodies or faces, or close-ups of dead bodies. The negative nature category consisted of thirty images that displayed close-ups of threatening animals that could potentially be dangerous, such as dogs, snakes, bears or spiders. The neutral category consisted of thirty images that displayed objects, people or scenes with no strong positive or negative connotations, such as household items, plants, buildings, or people walking on the street.

The negative social, negative physical and neutral images were solely selected from the IAPS database; negative nature images selected from the IAPS set were supplemented with images from the Kveraga et al. set. Image codes are reported in S1 Image codes. The images were selected from the IAPS set based on existing ratings of valence and arousal. Negative images were selected when the mean valence rating was below 4 and the mean arousal rating was above 5. Neutral images were selected when the mean valence rating was between 4 and 6 and the mean arousal rating was between 2 and 4. Analysis of the mean valence ratings demonstrated that negative images from all three categories were significantly more negative than the neutral images (all *p’s* < .001). Within the negative categories, negative nature images were significantly less negative than negative social and negative physical images (*p* < .001). Negative social and negative physical images did not differ in terms of valence (*p* = .45). Analysis of the mean arousal ratings demonstrated that negative images from all three categories were significantly more arousing than the neutral images (all *p’s* < .001). Within the negative categories, negative social, negative physical and negative nature images did not differ in terms of arousal (all *p’s* > .36).

#### Procedure

The paradigm was introduced as a study on exploring visual information. No mention of (morbid) curiosity was made. Participants were encouraged to participate seriously and to ignore social-desirable considerations in making their choice.

**Fig 1:**
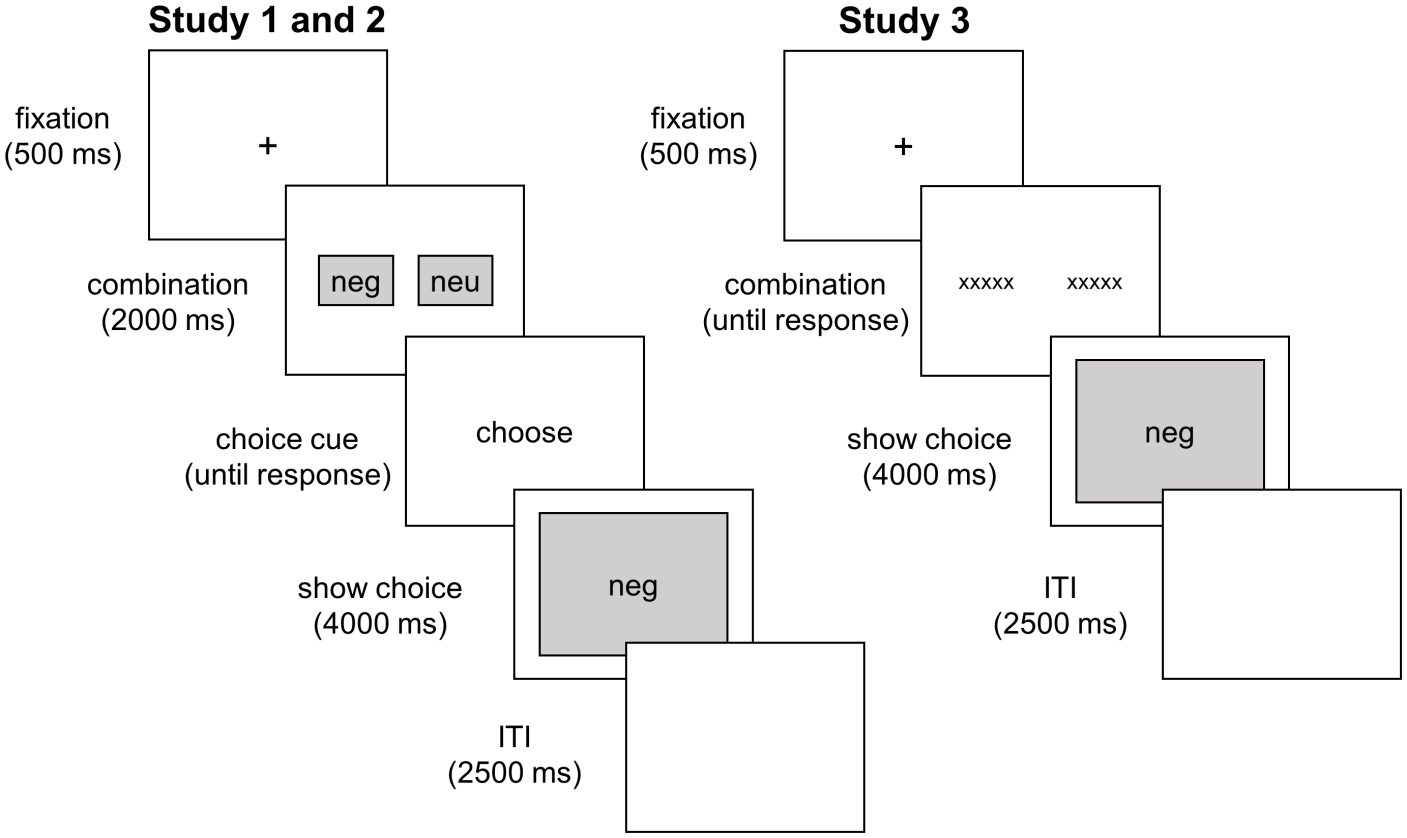
Overview of the choice paradigm.

The choice paradigm consisted of 60 trials and was presented with the stimulus presentation software E-prime. For an overview of the choice paradigm see Fig 1. Each trial started with a fixation cross presented for 500 ms, followed by a combination of two images (128 x 96 pixels or approximately 3.5 cm x 2.5 cm) presented in the middle of the screen. To make viewing time equal for each participant, the combination was presented for 2000 ms. Previous research has shown that a short exposure time of 250 ms is sufficient to categorize the valence of IAPS images [46]. Furthermore, participants are able to categorize whether an image contains a human or an animal when images are presented in very small format (similar to the size used in the present study) for 100 ms [47]. Based on these findings, it is assumed that presenting two small images on a computer screen for 2000 ms is sufficient for participants to process the valence of the images (but not long enough to take in all the details of the image). After 2000 ms the two small images were removed and the word “choice” was presented on the screen. Participants could choose with the ‘A’ (left) or the ‘L’ (right) key on the keyboard whether they wanted to see the left or the right alternative. When the participant made the choice, the chosen image appeared in full-screen format (1024 x 768 pixels) on the computer screen for 4000 ms. Trials were separated by a 2500 ms inter-trial-interval.

The choice paradigm consisted of the following combination conditions: negative social—neutral (10); negative physical—neutral (10); negative nature—neutral (10); negative social—negative physical (10); negative social—negative nature (10); negative physical—negative nature (10). The presentation of the different combination trials was fully randomized. The position of the images was counterbalanced (i.e., negative images were presented equally often on the left and on the right side). Moreover, the combination between specific images was varied in two different presentation lists, which alternated between participants. The statistical analyses regarding the choice between two negative images are reported in S1 Study; the mean choice proportions for these conditions are presented in Table 1.

After the choice task, participants were presented with all negative and neutral stimuli in full-screen format, and in random order and asked to rate these on interest, negativity, intensity and complexity. Each rating was made on a 0 (not interesting/negative/intense/complex) to 100 (very interesting/negative/intense/complex) continuous rating scale using a slider. Interest was explained to participants as reflecting the extent to which an image grabbed attention and was experienced as interesting. Negativity reflected to what extent an image was found negative. Intensity was explained as reflecting the extent to which an image evoked an activated feeling inside the body. Complexity was explained as reflecting the extent to which an image contained many details and/or was hard to understand.

#### Data analysis

Choice scores were expressed as the proportion chosen negative images within a particular combination condition (e.g., a choice score of .60 means that a participant chose a negative image in 60% of the presented trials). Across all studies in the present paper, the choice for negative images was significantly different from 0 (all *p’s* < .000001). As a strong test of morbid curiosity each results section reports one-sample *t*-tests with .5 as test value. Such a test examines whether participants *prefer* to view negative information over the alternative (i.e., whether participants chose a negative image in more than 50% of the trials). The question whether participants chose one particular category of negative information (i.e., social, physical and nature) more often than another was tested with a repeated measures ANOVA. One-sample *t*-tests and paired samples *t*-tests were tested against a threshold corrected for multiple comparisons with the Bonferroni-Holm method; comparisons that reached a *p* < .05 uncorrected threshold are explicitly noted.

To give some insight into how individuals varied in their choice for negative stimuli, S1 Individual variation reflects, for all studies and each choice condition, the range of choice proportions and the number of participants who chose the negative option in 0 to 59% of the trials and the number of participants who chose the negative option in 60% to 100% of the trials.

Boxplots did not indicate extreme outliers (> 3 * inter quartile range) in any of the studies presented in this paper. Across studies, Kolmogorov-Smirnov tests indicated that most variables reflecting choice were not normally distributed (-1.031 < skewness < .796; -1.383 kurtosis < .481). Since the samples of all studies exceeded 30 participants, analyses should be relatively robust against these deviations from normality [48, 49]. Nevertheless, all decisions regarding statistical significance based on one-sample *t*-tests presented in this paper were verified with bootstrapped *t*-tests and Wilcoxon signed rank tests. All correlation coefficients reported in the present paper (and the Supporting Information) are Spearman rhos. The data files containing the data discussed in the present paper are available on the Open Science Framework (https://osf.io/dpg2y/).

### Results

#### Choice for negative stimuli

As hypothesized, a one-sample *t*-tests demonstrated that, on average, participants preferred to view negative social images (M = .62; SD = .33) over neutral images, *t*(49) = 2.54, *p* = .014, *d* = .36. Negative physical images (M = .43; SD = .38) were chosen equally often as neutral images, *t*(49) = -1.39, *p* = .17. Negative nature images (M = .35; SD = .27) were chosen less often than neutral images, *t*(49) = -3.88, *p* < .001. A repeated measures analysis further demonstrated different choice behavior for the negative categories combined with a neutral alternative, *F*(2,98) = 19.71, *p* < .001, η^2^_p_ = .29. Negative social images were chosen significantly more often than both negative physical and negative nature images (*p’s* < .001; *d*_z_*’s* > .77).

#### Correlations between subjective ratings and choice

A correlation analysis examined the relationship between subjective ratings of interest, negativity, intensity and complexity and the proportion chosen negative images for the three negative–neutral choice conditions. All correlations are presented in Table 2. It is important to note that two correlations did not reach the pre-registered significance threshold. Choice for negative social images correlated with interest for negative social images, *ρ* = .39, *p* = .005; choice for negative physical images correlated with interest for negative physical images, *ρ* = .32, *p* = .026 (significant only at uncorrected threshold); choice for negative nature images correlated with interest for negative nature images, *ρ* = .30, *p* = .035 (significant only at uncorrected threshold). There were no significant (negative) correlations between the choice scores and interest ratings for neutral images, nor were there any correlations between the choice scores and subjective ratings of negativity, intensity or complexity.

**Table 2:** Correlations between choice proportions and subjective ratings.

		Interest	Negativity	Intensity	Complexity
Study 1	neg soc–neu	.39*	-.06	.00	-.12
	neg phy–neu	.32†	-.19	-.10	-.03
	neg nat–neu	.30†	-.04	.13	.12
Study 2	neg soc–neu soc	.34*	na.	na.	.11
	neg soc–pos soc	.37*	na.	na.	.16
	neg phy–neu phy	.44*	na.	na.	-.00
	neg phy–pos phy	.44*	na.	na.	.02
	neg nat–neu nat	.47*	na.	na.	.15
	neg nat–pos nat	.52*	na.	na.	.15
Study 3	neg soc–neu soc	.32*	.10	.18	na.
	neg soc–pos soc	.08	.05	.11	na.
	neg phy–neu phy	.57*	-.25	-.06	na.
	neg phy–pos phy	.53*	-.28†	-.06	na.

Note: Table reflects correlations between the proportion of negative options chosen in the different choice conditions and subjective ratings of negativity, intensity, interest and complexity of the corresponding negative image category.

* p < .05; corrected

^†^ p < .05; uncorrected.

### Discussion

Study 1 demonstrated that participants did not consistently avoid images portraying death, violence or harm, but instead chose to explore some of them. This exploration behavior is consistent with theoretical models of curiosity [10, 14, 20], and therefore interpreted as a reflection of “morbid curiosity”. In line with the “strong” hypothesis of morbid curiosity, Study 1 demonstrated that participants *preferred* to view social images that portrayed death, violence or harm over neutral images. Furthermore, and as hypothesized, morbid curiosity was more strongly expressed for negative social images than for the other categories of negative content. Physical harm images were chosen equally often as the neutral alternatives; negative nature images were chosen less often than the neutral alternatives.

Mean interest ratings for each negative category correlated positively with how often people chose images from that negative category during the choice paradigm (albeit tested against an uncorrected threshold). This finding is relevant because, following the assumption that interest and curiosity are closely related concepts [14], it supports the interpretation that choice for negative images reflects morbid curiosity. Nevertheless, it is important to note that because the interest ratings were made after the choice paradigm was performed, it is possible that choice behavior influenced the interest ratings (i.e., participants may have judged the images as more interesting because they chose them). In future research, an experimental design that would contrast an active choice condition with a random computer-generated choice condition may shed more light on this possibility. Interestingly, there was no correlation in the present dataset between choice for negative images and the judged intensity of those images. This finding is inconsistent with an explanation of morbid curiosity merely based on a motivation to seek out arousing experiences [1].

One possible interpretation of the findings from Study 1 is that the proportion chosen negative stimuli does not reflect a choice *for* a negative stimulus, but a choice *against* a neutral stimulus. Although there was no correlation between rated complexity and choice for negative images, the relatively higher visual complexity of the negative images as compared to the neutral images may have influenced choice. Moreover, previous research has shown that human content is overrepresented in high/arousal negative IAPS images as compared to low arousal/neutral IAPS images [50]. Thus, participants may have preferred social negative stimuli over neutral stimuli, because of the presence of interacting humans, and not because of the negative content per se. Therefore, Study 2 assessed choice behavior for negative images when the alternative choice was well matched in terms of content and visual configuration. Furthermore, Study 2 assessed choice behavior for negative images when the alternative choice was either relatively neutral or positive (e.g., pairing a negative social image with a positive social image).

Study 2 tested the hypothesis that participants would prefer to view negative social images over neutral social images. Furthermore, the study also tested the hypothesis that, overall, the proportion chosen negative social images would be significantly higher than the proportion chosen negative physical and negative nature images. Regarding the choice between negative physical images and neutral physical images, it was hypothesized that participants would not prefer negative physical images. The study did not test specific hypotheses regarding negative nature stimuli and for the choice between negative stimuli and positive alternatives. Finally, based on the findings of Study 1 regarding the interest ratings, the study tested the hypothesis that mean interest ratings for the images in each negative category would correlate positively with the proportion of chosen negative images of that category.

## Study 2

### Method

#### Participants

Fifty-four students (40 females; Mean age = 21.6; SD = 2.72) from the University of Amsterdam signed the informed consent form. The study was approved by the Ethics Review Board of the Psychology Department at the University of Amsterdam (2014-SP-3912). Prior to analysis, four participants were excluded from the sample. Three participants were excluded because of missing data; one participant was excluded because the experimenter doubted the sobriety of the participant. All other details were identical to Study 1.

#### Design

The factor *category* (social vs. physical vs. nature) reflected whether the target and the alternative had social, physical or nature content. The factor *combination* (neutral vs. positive) reflected whether the non-target alternative was neutral or positive. Both factors were varied within participants.

#### Materials

The negative images were a subset (28 items for each category) of the images described in the method section of Study 1. For each negative image a matching image was found that was highly similar in terms of the number of people or animals displayed or the type of body part displayed. Furthermore, the stimuli were closely matched in terms of visual configuration (e.g., orientation of bodies) and background information (e.g., indoors or outdoors). Half of the negative social images (*n* = 14) were matched with relatively neutral social images displaying neutral social situations, such as people waiting at a bus stop, conversing in meetings, or packing groceries in the supermarket. The other half (*n* = 14) was matched with relatively positive social images displaying positive social situations, such as people lying on the beach, crowd surfing at a concert, or having a family dinner. Half of the negative physical images (*n* = 14) were matched with relatively neutral physical images displaying neutral body parts up close, such as a relaxed arm, a body in a yoga position, or a neutral face. The other half (*n* = 14) was matched with relatively positive physical images displaying body parts in a positive context, such as a back being massaged, two hands on a pregnant belly or a neck with a beautiful necklace. Finally, half of the negative nature images (*n* = 14) were matched with relatively neutral nature images displaying common animals, such as a bird, a dog or a badger. The other half (*n* = 14) was matched with relatively positive nature images displaying exotic or cute animals, such as butterflies, a kitten or a puppy.

To verify that the images were relatively well matched in terms of low-level visual features, two summary statistics (i.e., *feature energy* and *spatial coherence*) were calculated and compared across image conditions. Most importantly, all conditions paired in the choice paradigm (e.g., negative social vs. neutral social) were similar in terms of spatial coherence (i.e., a feature reflecting scene fragmentation that is associated with decision-making processes; [51]). All details of the visual feature analyses are presented in S2 Study.

The neutral and positive images were selected from the IAPS database [44], the Nencki Affective Picture System database (NAPS; [52]) and the internet. Image codes and images selected from the internet are available on request from the author.

#### Procedure

The paradigm was in all aspects identical to Study 1. After the choice task, participants rated all negative, positive and neutral stimuli on interest and complexity on a 0 to 100 continuous rating scale using a slider. A full analysis of the subjective ratings is presented in S2 Study.

### Results

#### Choice for negative stimuli

In line with the hypothesis, participants preferred to view negative social images over neutral social images (*M* = .58; *SD* = .28), *t*(49) = 2.04, *p* = .047, *d* = .29. Note, however, that this effect did not reach significance at the Bonferroni-Holm corrected threshold. Negative social images were chosen less often than positive social images (*M* = .43; *SD* = .25), *t*(49) = -2.04, *p* = .046, *d* = .28, also only when testing against an uncorrected threshold. In contrast to the hypothesis, negative physical images were chosen less often than neutral physical images (*M* = .36; *SD* = .32), *t*(49) = -3.08, *p* = .003, *d* = .44. Furthermore, negative physical images were chosen less often than positive physical images (*M* = .35; *SD* = .29), *t*(49) = -3.79, *p* < .001, *d* = .52. Negative nature images were chosen less often than neutral nature images (M = .39; SD = .25), *t*(49) = -3.06, *p* = .004, *d* = .44, and positive nature images (M = .30; SD = .25), *t*(49) = -5.70, *p* < .001, *d* = .81.

A repeated measures analysis demonstrated different choice behavior for the three negative categories, *F*(2,98) = 13.65, *p* < .001, η^2^_p_ = .22. As hypothesized, a main effect of category demonstrated that, overall, negative social images (M = .51; SE = .036) were chosen more often than negative physical (M = .35; SE = .042) and negative nature images (M = .34; SE = .033). In addition, there was a main effect of valence, *F*(1,49) = 45.94, *p* < .001, η^2^_p_ = .48. Negative images combined with a neutral alternative (M = .44; SE = .034) were chosen more often than negative images combined with a positive alternative (M = .36; SE = .031). Finally, there was an interaction between category and valence, *F*(2,98) = 10.06, *p* < .001, η^2^_p_ = .17. Follow-up paired samples *t*-tests demonstrated that the proportion chosen negative images in the negative social—neutral social condition was significantly higher than in all other combination conditions (*p’s* < .001; *d*_z_*’s* > .66). See Table 1 for an overview of all comparisons.

#### Correlations between subjective ratings and choice

Replicating Study 1, the interest ratings for the images in each negative category correlated positively with the proportion chosen negative images of that category (all *ρ’s* > .34; *p’s* < .018, see Table 2). Consistent with the findings from Study 1, there were no significant (negative) correlations between the choice scores and interest ratings for neutral images or positive images, nor were there any correlations with the complexity ratings.

### Discussion

Study 2 examined whether participants chose to view images portraying death, violence or harm when those negative images were combined with neutral or positive alternatives that were similar in content. In line with the hypotheses, curiosity was most strongly expressed for negative social images; this category was preferred over neutral social images with similar low-level visual properties, and chosen significantly more often than the two other negative categories. Study 2 clearly demonstrated that choice for negative social images changed depending on the valence of the alternative (i.e., it dropped from 58% to 43% depending on whether it was paired with a neutral or positive alternative respectively). In contrast to the findings of Study 1, images displaying physical harm were chosen less often than the alternative, irrespective of whether the alternative was neutral or positive. Images displaying natural threat were also chosen less often than the neutral or positive alternative. Finally, as in Study 1, there was a positive correlation between interest ratings for the images in each negative category and the proportion of chosen negative images of that category.

## Study 3

In the first two studies, participants could choose between two visual cues that were presented briefly (for 2 seconds) and in small format (3.5 cm by 2.5 cm). Although this format was chosen deliberately to evoke curiosity, at the same time it may have made it difficult for participants to see what was going on in an image. In Study 1 the negative images were more visually complex than the neutral images, and as a consequence, participants may have chosen the negative images not because of their negativity, but because of their visual complexity. Study 2 provided evidence against this interpretation by demonstrating similar choice behavior when images were well matched in terms of content and visual configuration (please see S2 Study for more details).

Although these findings suggest that people indeed choose to view negativity, rather than simply the most visually complex stimulus, another way of countering the possible role of visual complexity would be to demonstrate similar choice proportions for negative images when that choice is not based on the visual characteristics of stimuli. Therefore, Study 3 aimed to replicate the findings of the first two studies with a paradigm that presented verbal cues. Participants were presented with short verbal descriptions of images (e.g., “*firemen carry a dead woman*” vs. “*friends carry a smiling friend*”; see Fig 1) and were asked to choose which of the corresponding images they wanted to view. These verbal cues eliminate the issue of visual complexity, and mimic a common way in which people are confronted with information that may spike morbid curiosity in reality, such as headlines in a newspaper or image links on the internet (e.g., Facebook, YouTube).

Study 3 tested the hypothesis that participants would prefer to view negative social images over neutral social images, but not over positive social images. Furthermore, as in Study 1 and 2, the hypothesis was tested that social negative images would be chosen more often than physical negative images. Regarding the choice between negative physical images and the alternatives, it was hypothesized that participants would not show a preference for negative physical images. Nature images were not included in Study 3, because it was impossible to generate non-repetitive verbal descriptions of nature images. After the choice task, participants rated all images on negativity, intensity and interest. As in Study 1 and 2 the hypothesis was tested that choice for negative stimuli would correlate positively with interest ratings of the corresponding images at a later time.

### Method

#### Participants

Seventy-three students (39 females; Mean age = 21.5; SD = 2.74) from the University of Amsterdam signed the informed consent form. The study was approved by the Ethics Review Board of the Psychology Department at the University of Amsterdam (2015-SP-4075). The study aimed to sample an equal number of male and female participants to explore possible gender differences in curiosity for negative information [21]. These analyses can be found in S1 Gender Differences. All other details were identical to Study 1.

#### Design

The factor *category* (social vs. physical) reflected whether the target and the alternative had social or physical content. The factor *combination* (neutral vs. positive) reflected whether the non-target alternative was neutral or positive. Both factors were varied within participants.

#### Materials

The images (28 per category) were the same as the images used in Study 2. For each image a short description was written that described the content of the image. For example, a negative social image was described as “*a wounded boy is carried away from disaster*”; a neutral physical images was described as “*group of women is resting during a conference*”; a positive physical image was described as “*partying people are carrying a crowd surfer*”; a negative physical image was described as “*chopped-off hand and wrist*”; a neutral physical images was described as “*a spread-out hand*”; a positive physical image was described as “*large belly of a pregnant woman*”. The descriptions of the negative images did not differ in length (in characters) from the descriptions of the neutral and positive images (all *p’s* > .28).

Half of the negative social descriptions (14) were paired with neutral social descriptions; the other half (14) were paired with positive social descriptions. Half of the negative physical descriptions (14) were paired with neutral physical descriptions; the other half (14) were paired with positive physical descriptions. Study 3 did not include nature stimuli.

The adapted choice paradigm utilizing descriptions as cues consisted of 56 trials presented with the stimulus presentation software E-prime (see Fig 1). Each trial started with a fixation cross presented for 500 ms, followed by two descriptions presented side by side on the screen (Courier New, 24 pt). The descriptions remained on the screen until the participants made a choice. Participants could choose to view the image described by the left description by pressing the ‘A’ (left) key or the image described by the right description by pressing the ‘L’ (right) key on the keyboard. When the participant had made the choice, the chosen image appeared in full-screen format (1024 x 768 pixels) on the computer screen for 4000 ms. Trials were separated by a 2500 ms inter-trial-interval.

After the choice task, participants rated all negative, positive and neutral images on interest, intensity and complexity on a 0 to 100 continuous rating scale using a slider. Due to time constraints, only a subset of the sample (*n* = 55) completed the subjective ratings. For the sake of brevity, the analyses of the subjective ratings are presented in S3 Study. The correlations with choice are presented in Table 2.

### Results

#### Choice for negative stimuli

As hypothesized, participants chose negative social images more often than neutral social images (M = .67; SD = .25), *t*(72) = 5.78, *p* < .001, *d* = .68. In contrast to Study 2, negative social images were chosen equally often as positive social images (M = .47; SD = .26), *t*(72) = -.98, *p* = .33. Negative physical images were chosen less often than neutral physical images (M = .35; SD = .28), *t*(72) = -4.63, *p* < .001, *d* = .54. Furthermore, negative physical images were chosen less often than positive physical images (M = .32; SD = .24), *t*(72) = -6.56, *p* < .001, *d* = .77.

A repeated measures analysis demonstrated different choice behavior for the two negative categories, *F*(1,72) = 159.21, *p* < .001, η^2^_p_ = .69. As hypothesized, overall, negative social images (M = .57; SE = .028) were chosen more often than negative physical images (M = .33; SE = .028). In addition, there was a main effect of valence, *F*(1,72) = 70.70, *p* < .001, η^2^_p_ = .50. Negative images combined with a neutral alternative (M = .51; SE = .028) were chosen more often than negative images combined with a positive alternative (M = .39; SE = .027). Finally, there was an interaction between category and valence, *F*(1,72) = 32.17, *p* < .001, η^2^_p_ = .31. Follow-up paired samples *t*-tests demonstrated that the proportion chosen negative images in the negative social—neutral social condition was significantly different from all other combination conditions (*p’s* < .001; *d*_z_*’s* > 1.2). See Table 1 for an overview of all comparisons.

### Discussion

Study 3 examined choice for images portraying death, violence or harm with verbal descriptions instead of visual information as choice cues. As hypothesized, people chose to view negative social images more often than negative physical images. Furthermore, and in line with Study 1 and 2, negative social images were preferred over neutral social alternatives, but not over positive social alternatives. In contrast to Study 2, negative social images were chosen equally often as positive social alternatives. And finally, negative physical images were chosen less often than the neutral and positive physical alternatives. Thus, participants made largely similar choices as in Study 1 and 2, when they based their choice on verbal descriptions of negative images.

## General discussion

The first aim of the present paper was to examine, in a carefully controlled experimental setting, whether people deliberately choose to view images that portray death, violence or harm over a non-negative alternative. The second aim was to explore whether choice behavior was affected differently by the *type* of negative information. With regard to these two aims, the present studies consistently demonstrated that participants chose to view images that portrayed death, violence or harm. Most notably, and in line with a “strong” hypothesis regarding morbid curiosity, participants *preferred* to view social negative content when the alternative was neutral (i.e., above and beyond the fact that participants opted to view negative images at all, they chose social negative images significantly more often than the neutral alternatives). When the alternative was relatively positive participants chose social negative images in about 40–50% of the trials. Furthermore, across all studies, participants chose to view images portraying death, violence or harm within a social context more often than images portraying graphic physical harm or attacking animals. This indicates that the type of negative information affects the extent to which participants display morbid curiosity.

These findings counter the assumption that there is a fundamental link between negative stimuli and avoidance motivation [41]. People deliberately choose to view highly intense negative stimuli displaying death, mutilation, and violent social conflict. Based on theoretical models that define curiosity in terms of approach behavior or exploration [10, 14, 20], the choice behavior observed in the present studies is interpreted as a reflection of morbid curiosity [1]. This interpretation is supported by the finding that choice behavior correlated positively with subjective ratings of interest, a concept closely related to curiosity [14].

Before this paper will discuss what may drive curiosity for negative images in general and social images in particular, there are a few important issues to discuss concerning the interpretation of the present findings. First of all, the aim of this paper was to test whether participants would choose to view images that portray death, violence or harm when given an easy opportunity to avoid them. To examine this, the present studies utilized neutral and relatively mild positive stimuli as alternatives. Currently, it is an open question whether alternative stimulus categories exist that would “nullify” the choice for negative information. For example, as an avenue for future research, it would be interesting to present choices between highly intense negative images and highly intense positive images portraying, for example, erotic acts, extreme sports, or highly surprising events.

Secondly, even though the averaged choice proportions suggest an overall morbid curiosity effect, there was individual variation in how often people chose to view negative material. As can be seen in S1 Individual variation, some participants never chose to view a negative image; some participants chose negative images in a minority of the trials; some participants chose negative images in the majority of the trials, and some participants always chose the negative image. One potential factor that may explain these individual differences is coping potential [13]; people who chose to avoid negative images may have made predictions about their inability to cope with this material.

Third, there were no correlations between choice for negative images and subjective ratings of intensity. This is an interesting finding because a sensation seeking account of morbid curiosity [1] proposes that people seek out negative events because of the arousal or intense sensations that these may evoke. The intensity ratings collected in the present studies were not consistent with this proposal: higher choice proportions for negative images were not associated with higher intensity ratings. Regarding negativity, only Study 3 suggested that higher choice proportions for negative physical images (based on verbal cues) were associated with lower negativity ratings of negative physical images. Nevertheless, because this result was only found to be significant once, at an uncorrected threshold, it will not be further discussed. None of the studies demonstrated a negative (or positive) correlation between choice and rated negativity for social negative images. This finding is consistent with previous research that demonstrated no relationship between interest and pleasantness when people judged disturbing and non-disturbing art [18].

Fourth, the present studies found no correlation between choice for negative images and judged complexity of those images. This is an important finding since complexity is seen as a possible drive of curiosity [13, 20]. With regards to visual complexity, Study 2 demonstrated that participants preferred negative social images over neutral social images even when those images were relatively well matched in terms of visual characteristics. The role of visual complexity was circumvented in Study 3, in which the preference for negative social images over neutral social images was replicated when participants made choices based on verbal descriptions of the images. Nevertheless, even though the present studies found no evidence that complexity is an important factor in choice for negativity, it must be noted that not all forms of complexity were examined. For example, it is an open question whether negative stimuli, in particular those with social content, have a higher level of semantic or narrative complexity, which may influence curiosity.

### What drives morbid curiosity?

So far morbid curiosity has mainly been explained in terms of a sensation seeking motivation [1]. Applying such an interpretation to the present findings would imply that people choose to view negative images because they seek out the sensations or experiences that they expect will be evoked by the selected stimulus [53]. An alternative, though not mutually exclusive, interpretation is that people choose negative images because of an epistemic motivation. In other words, people may be curious about negative stimuli because these stimuli allow people to acquire knowledge about the world [20]. This interpretation is consistent with models that characterize curiosity as a “drive state for information” ([54]; p. 450), as a state characterized by a motivation to explore information [10], or as a state characterized by a motivation to close an “information gap” [11].

If we consider the possibility that curiosity is about the informational value of a stimulus, then what do images that portray death, violence or harm have to offer in informational terms? According to Baumeister and colleagues [55] negative events have greater psychological impact (e.g., better memory, more cognitive elaboration) than neutral or positive events, because it is evolutionary adaptive to be sensitive to negative information. Another interpretation is that learning about emotional events (both positive *and* negative) may expand the conceptual system that according to some emotion models is crucial in the experience and regulation of emotional states [56]. Thus, people may explore stimuli that portray death, violence or harm because it gives them handholds that are useful in dealing with future negative situations.

Another relevant suggestion made by Unkelbach and colleagues [57] is that positive information is very much alike, whereas negative information is often uniquely negative. Translating this to the current findings, this means that people may choose stimuli that portray death, violence or harm because they deviate from the norm, and thus are associated with a relatively strong gain in information. This is in line with suggestions from other authors that people are curious about stimuli that present a relatively rare source of information [20] or a relatively uncertain or contradictory source of information [18, 19].

In addition, it is also relevant to address what negative *social* stimuli, specifically, have to offer in informational terms. Interestingly, Kashdan and Silvia [14] suggest that curiosity for people and social situations may offer psychological and social benefits. It is important to note, however, that this suggestion is made within the context of discussing curiosity as a positive state, whereas the present paper targets curiosity for *negative* social information. That said, Kashdan and Silvia’s argument that social situations are often ambiguous and challenging can be applied to negative social situations, and may explain why this type of information evokes exploratory behavior. Moreover, social information may have a relatively high learning potential. Developmental studies, for example, show that young infants have a preference for social stimuli (see for an overview [58]). Applying these interpretations to morbid curiosity, it may be the case that the exploration of negative social information helps an individual to acquire and encode important knowledge about the social environment.

Furthermore, an image of a negative event placed within a social context may evoke a more complex narrative [59] as compared to an image that portrays a decontextualized attacking animal or a decontextualized portrayal of physical harm. Recent research within literature science has shown that negative narratives can be attractive [60]. Furthermore, the narrative suggested by a negative social scene may evoke questions, such as What happened? What are the relationships between these people? What will happen after this scene? Participants may choose the negative social images more often as compared to the images portraying graphic physical harm or attacking animals, because they want to find answers or clues to these questions (and close the information gap; [11]).

Finally, the urge to inform oneself about the details of a negative social situation may not only be driven by an information-seeking motivation, but potentially also by a motivation to experience empathy or sympathy for the people portrayed in the images (see for a similar point [50, 61]). Indeed, some studies on empathy have used stimuli with similar content as the negative social images used in the present studies (e.g., [37, 38, 62]). Moreover, people may experience a sense of moral responsibility to inform oneself about the reality of other people’s suffering [63]. The question how different motivations to empathize might influence people’s choice to expose themselves to the negative experiences of others is an important avenue for future research (see also [64]).

### Conclusion

The present paper advances new insights into the phenomenon of morbid curiosity and implies that there is a dynamic relationship between negative stimuli and behavior. Images that portray death, violence or harm are not always avoided, but can evoke curiosity driven approach behavior. These findings expand theoretical models of curiosity that have focused mainly on the reward value of positive stimuli (e.g., [65]; see for a similar point [10, 18, 19]). Furthermore, these findings connect to current models of emotion that argue for individual and situational diversity in how affective stimuli are experienced [56, 66].

In practical terms, the present findings also have methodological implications. First of all, the present paper presents a paradigm that provides a behavioral index of (morbid) curiosity that can be used in future studies to test situational and contextual moderators of curiosity and interest-related constructs. Furthermore, with some adaptations the paradigm is suited to be combined with physiological and neurological measures to understand how curiosity for negative information (or other types of information) is represented in the brain and body. Another important methodological implication concerns the fact that all negative images that were used in the present paradigm were selected from standardized image sets, such as the IAPS database [44]. These same images are routinely used in affective science to study a variety of emotional reactions, such as fear, disgust, arousal or negative affect. When using this kind of material it may be important for affective scientists to take into consideration that some of the participants in the sample may actually *want* to view images that portray death, violence or harm.

In short, the present series of studies provide new insights about the occurrence of morbid curiosity, a largely neglected topic of investigation. Although many questions remain unanswered, and more research is needed, the present paper aims to put this phenomenon back on the research agenda of affective science.

## Supporting information

S1 Pilot study Supporting Information regarding the Pilot Study. (DOCX)

S1 Image codes Image codes for all images used in Study 1. (DOCX)

S1 Individual variation Supporting Information regarding individual variation across studies. (DOCX)

S1 Gender differences Supporting Information regarding gender differences in the Pilot Study and Study 3. (DOCX)

S1 Study Supporting Information regarding Study 1. (DOCX)

S2 Study Supporting Information regarding Study 2. (DOCX)

S3 Study Supporting Information regarding Study 3. (DOCX)
