# Supplementary material for: Choosing the negative: A behavioral demonstration of morbid curiosity
Source: PLoS One. 2017 Jul 6;12(7):e0178399. doi: 10.1371/journal.pone.0178399 (PMC5500011; doi:10.1371/journal.pone.0178399)
Supplement: S1 Pilot study — (DOCX) [file pone.0178399.s001.docx]

**Supporting Information Pilot Study**

*Participants and Procedure.* Thirty-eight students (21 females; Mean age = 21.9; SD = 2.87) from the University of Amsterdam signed the informed consent form. No participants were excluded from the sample. All details about the design, materials and procedure of the choice task were identical to Study 1.

After the choice task participants filled in an explorative, newly developed questionnaire, that measured self-reported tendencies to engage with negative information or events in daily life (e.g., “*If the police puts video footage on Youtube of someone who is beaten up, because they are looking for the perpetrators, then I will view the footage.*”; “*If a book holds too many details about violence, then I will stop reading*”). All questions in this questionnaire are presented on page 3 of this Supporting Information file. In addition, participants filled in the Arnett Inventory of Sensation Seeking (AISS; Arnett, 1994), Interpersonal Reactivity Index (IRI; Davis, 1983), Need for Cognition Scale (NFCS, Cacioppo & Petty, 1982) and the Behavioral Activation and Behavioral Inhibition Scale (BISBAS; Carver & White, 1994).

*Choice for negative stimuli.* One-sample *t*-tests demonstrated that, on average, participants preferred to view negative social images (M = .70; SD = .26) over neutral images, *t*(37) = 4.80, *p* < .001, *d* = .78. Negative physical images (M = .53; SD = .29) were chosen equally often as neutral images, *t*(37) = .605, *p* = .55. Negative nature images (M = .47; SD = .28) were also chosen equally often, *t*(37) = -.588, *p* = .56. A repeated measures analysis that compared choice for the three different negative categories combined with a neutral alternative, demonstrated a main effect of category, *F*(2,74) = 16.71, *p* < .001, η²_p_= .31. Follow-up paired samples *t*-tests demonstrated that negative social images were chosen significantly more often than both negative physical and negative nature images (*p’s* < .001; *d_z_*‘s > .82).

When choosing between two negative images, participants preferred to view negative social images (M = .66; SD = .25) over negative physical images, *t*(37) = 4.10, *p* < .001, *d* = .66, and negative social images (M = .64; SD = .23) over negative nature images, *t*(37) = 3.69, *p* < .01, *d* = .60. Negative physical and negative nature images were chosen equally often, *t*(37) = -.805, *p* = .43.

*Choice behavior and questionnaires.* A non-parametric correlation analysis examined the relationship between the collected self-report measures and the proportion chosen negative images for the three negative – neutral choice conditions. All reported coefficients are Spearman’s rho (*ρ*).

Choice for negative social images was correlated with the novelty seeking subscale (α = .56), *ρ* = .38, *p* = .019, and the intensity seeking subscale (α = .56), *ρ* = .61, *p* < .001, of the AISS. Choice for negative physical images also correlated with novelty seeking, *ρ* = .41, *p* = .011, and intensity seeking, *ρ* = .52, *p* < .01. Choice for negative nature images did not correlate with the AISS subscales. These results are in line with previous work demonstrating relationships between sensation seeking and self-reported viewing of horror movies and violence (Zuckerman & Litle, 1986; Aluja-Fabregat, 2000; Hoffner & Levine, 2005).

Furthermore, both choice for negative social (*ρ* = .64, *p* < .001) and negative physical images (*ρ* = .58, *p* < .001) correlated significantly with the self-reported tendency to engage with negative information or events in daily life (α = .60). Although this questionnaire is not validated, and hence the results should be interpreted with caution, these results seem to suggest that the choice behavior measured by the present paradigm is similar to how participants behave outside of a lab setting.

Finally, there was a significant correlation between choice for negative social images and the BAS-reward subscale (α = .67), *ρ* = .41, *p* = .010, and the BAS-funseeking subscale, (α = .67), *ρ* = .42, *p* < .01, of the BIS/BAS. Furthermore, there was a significant correlation between choice for negative social images and self-reported need for cognition (α = .67), *ρ* = .35, *p* = .031. There were no significant correlations between any of the other choice variables, and the BAS-drive subscale, the BIS subscale, or the subscales from the IRI, although some correlations approached significance (see Table 1 in this Supporting Information file).

Table 1. Correlations between choice and self-report subscales Pilot Study.

|  | Choice for social negative | Choice for physical harm negative | Choice for nature negative |
| --- | --- | --- | --- |
| ASS-novelty | .38* | .41* | .28† |
| ASS-intensity | .61** | .52** | .23 |
| Morbid curiosity in daily life | .64** | .58** | .22 |
| BIS-scale | -.08 | -.07 | -.13 |
| BAS-reward | .41* | .27 | .16 |
| BAS-drive | .13 | -.12 | .06 |
| BAS-funseeking | .42** | .26 | .20 |
| Need for Cognition | .35* | .30† | .22 |
| IRI Perspective | -.19 | -.32† | .01 |
| IRI Concern | -.28† | -.31† | -.14 |
| IRI Fantasy | -.20 | -.26 | -.18 |
| IRI Distress | -.09 | -.05 | -.02 |

† *p* < .10

* *p* < .05

** *p* < .01

**Morbid curiosity in daily life questionnaire**

We would like to present you with a series of statements about different forms of collecting information. Please note on a scale from 1 (Not at all applicable) to 7 (Strongly applicable) to what extent these statements are applicable to you.

1. When a large disaster, terrorist attack or other shocking event has taken place, then I like to keep myself updated on all the details.
2. I think it would be interesting to visit an exposition that displays ancient mummified human bodies.
3. If the police puts video footage on Youtube of someone who is beaten up, because they are looking for the perpetrators, then I will view the footage.
4. I believe that art can also be impressive when it has an dark, shocking or provoking theme.
5. I think that media companies should not involve themselves with the production of explicitly violent television shows. (reverse scored)
6. I think it is important that newspapers publish photographs of shocking events, because this is often the best way to transfer the intensity of those events.
7. If a book holds too many details about violence, then I will stop reading. (reverse scored)
8. I like to watch movies or shows in which the main character carries out forensic research to solve gruesome crimes.
9. I find it inconceivable that people have the tendency to watch when an accident has happened. (reverse scored)

**References**

Aluja-Fabregat, A. (2000). Personality and curiosity about TV and films violence in

adolescents. *Personality and Individual Differences, 29,* 379-392.

Arnett, J. (1994). Sensation seeking: A new conceptualization and a new scale. *Personality*

*and Individual Differences, 16,* 289-296.

Cacioppo, J.T., & Petty, R.E. (1982). The need for cognition. *Journal of Personality and Social Psychology, 42*, 116-131.

Carver, C. S. & White, T. L. (1994). Behavioral inhibition, behavioral activation, and

affective responses to impending reward and punishment: The BIS/BAS scales. *Journal of Personality and Social Psychology, 67,* 319-333.

Davis, M. H. (1983). Measuring Individual Differences in Empathy: Evidence for a

Multidimensional Approach. *Journal of Personality and Social Psychology, 44, 1,*

113-126.

Hoffner, C.H., & Levine, K.J. (2005). Enjoyment of mediated fright and violence: a meta-analysis. *Media Psychology, 7*, 207-237.

Zuckerman, M. & Litle, P. (1986). Personality and curiosity about morbid and sexual events.

*Personality and Individual Differences, 7,* 49-56.
