# Supplementary material for: Choosing the negative: A behavioral demonstration of morbid curiosity
Source: PLoS One. 2017 Jul 6;12(7):e0178399. doi: 10.1371/journal.pone.0178399 (PMC5500011; doi:10.1371/journal.pone.0178399)
Supplement: S1 Image codes — (DOCX) [file pone.0178399.s002.docx]

**Supporting Information Image codes**

Table. Image codes for all images used in Study 1.

| Category | Images codes from the IAPS set and Kveraga set |
| --- | --- |
| Social negative images (30) | 2141, 2683, 2688, 2691, 2799, 3216, 3530, 6022, 6212  6313, 6520, 6831, 8485, 9050, 9163, 9250, 9252, 9253  9254, 9400, 9410, 9413, 9419, 9427, 9428, 9429, 9433  9435, 9900, 9921 |
| Physical negative images (30) | 2981, 3001, 3005, 3015, 3016, 3017, 3019, 3030, 3060, 3061, 3064, 3071, 3100, 3101, 3103, 3130, 3160, 3168, 3195, 3212, 3213, 3250, 3400, 7361, 9043, 9405, 9420, 9490, 9500, 9635_1 |
| Nature negative images (30) | 1019, 1050, 1051, 1052, 1090, 1110, 1111, 1113, 1120, 1200, 1201, 1205, 1220, 1274, 1300, 1301, 1525, 1932, threat_direct001T, threat_direct01N, threat_direct021T, threat_direct05N, threat_direct078N, threat_direct09N, threat_direct14N, threat_direct15N, threat_direct16N  threat_direct59N, threat_direct68N, threat_direct70N |
| Neutral images (30) | 2038, 2190, 2191, 2206, 2235, 2397, 2440, 2480, 2570  2593, 2594, 2840, 2890, 5395, 5471, 5510, 5535, 5731  5731 ,5740, 5740, 7001, 7009, 7019, 7034, 7035, 7036  7038, 7041, 7052 |

Note. Image codes are taken from the International Affective Pictures System database (IAPS; Lang, Bradley & Cuthbert, 2008) and the image set developed by the Kveraga lab (<http://www.kveragalab.org/stimuli.html>; Kveraga, Boshyan, Adams, Mote, Betz, Ward, Hadjikhani, Bar & Barrett, 2015).
