# Supplementary material for: Choosing the negative: A behavioral demonstration of morbid curiosity
Source: PLoS One. 2017 Jul 6;12(7):e0178399. doi: 10.1371/journal.pone.0178399 (PMC5500011; doi:10.1371/journal.pone.0178399)
Supplement: S1 Individual variation — (DOCX) [file pone.0178399.s003.docx]

**Supporting Information Individual variation**

Table. Overview of individual variation.

|  |  | Range in choice (min – max) | 0% to 59%  negative chosen | 60% to 100% negative chosen | Total n |
| --- | --- | --- | --- | --- | --- |
| Pilot | neg soc – neu | 10% - 100% | 9 (24%) | 29 (76%) | 38 |
|  | neg phy – neu | 0% - 100% | 21 (55%) | 17 (45%) | 38 |
|  | neg nat – neu | 10% - 100% | 23 (60%) | 15 (40%) | 38 |
| Study 1 | neg soc – neu | 0% - 100% | 16 (32%) | 34 (68%) | 50 |
|  | neg phy – neu | 0% - 100% | 33 (66%) | 17 (34%) | 50 |
|  | neg nat – neu | 0% - 90% | 37 (74%) | 13 (26%) | 50 |
| Study 2 | neg soc – neu soc | 0% - 100% | 25 (50%) | 25 (50%) | 50 |
|  | neg soc – pos soc | 0% - 93% | 37 (74%) | 13 (26%) | 50 |
|  | neg phy – neu phy | 0% - 100% | 38 (76%) | 12 (24%) | 50 |
|  | neg phy – pos phy | 0% - 100% | 39 (78%) | 11 (22%) | 50 |
|  | neg nat – neu nat | 0% - 93% | 40 (80%) | 10 (20%) | 50 |
|  | neg nat – pos nat | 0% - 93% | 42 (84%) | 8 (16%) | 50 |
| Study 3 | neg soc – neu soc | 0% - 100% | 23 (31%) | 50 (69%) | 73 |
|  | neg soc – pos soc | 0% - 100% | 51 (70%) | 22 (30%) | 73 |
|  | neg phy – neu phy | 0% - 100% | 62 (85%) | 11 (15%) | 73 |
|  | neg phy – pos phy | 0% - 100% | 63 (86%) | 10 (14%) | 73 |

Note. Table reflects individual variation in choice in Studies 1 through 3 for descriptive purposes. For each choice condition the table presents the range in choice proportions within the sample and the number of participants (percentage in parentheses) that chose the negative option in 0 to 59% of the trials or that chose the negative option in 60% to 100% of the trials (majority).
