# Supplementary material for: Choosing the negative: A behavioral demonstration of morbid curiosity
Source: PLoS One. 2017 Jul 6;12(7):e0178399. doi: 10.1371/journal.pone.0178399 (PMC5500011; doi:10.1371/journal.pone.0178399)
Supplement: S1 Gender differences — (DOCX) [file pone.0178399.s004.docx]

**Supporting Information Gender Differences**

Because previous research has indicated gender differences in curiosity for negative information (e.g., Zaleski, 1984), the effect of gender on choice for negative stimuli was examined exploratively in the pilot study and Study 3.

*Pilot Study*. Although the pilot study did not specifically sample for an equal gender division, the final sample did allow for an exploration of differences in choice behavior between males (*n* = 21) and females (*n* = 17). A repeated measures analysis with choice for the three different negative categories as a within factor and gender as a between factor, demonstrated a main effect of gender, *F*(1,36) = 6.22, *p* = .017, η²_p_ = .15. Overall, males chose to view negative images more often (*M* = .65; *SE* = .048) than females (*M* = .47; *SE* = .053). There was no interaction between *gender* and *category*, *F* < 1.

*Study 3*. Study 3 deliberately sampled a similar number of male (*n* = 34) and female (*n* = 39) participants to explore gender differences. A repeated measures analysis of the choice proportions that included gender as a factor did not demonstrate a significant main effect of gender or an interaction with gender (all *F’s* < 1).

*Discussion*. These two studies provide somewhat mixed results regarding sex differences in choice behavior. In the pilot study, where participants were presented with visual cues, males chose the negative option more often than females. In Study 3, where participants were presented with verbal cues, there was no difference between males and females in terms of choice behavior. One possible reason for this discrepancy may be that females respond stronger to visual portrayals of negativity than males (Bradley, Codispoti, Sabatinelli & Lang, 2001) and that therefore they make different decisions following visual cues, but not verbal cues. Nevertheless, previous research on sex differences in emotional responding has been mixed (Hamann & Canli, 2004; Kring & Gordon, 1998), and therefore it is advised to replicate and further expand on the present findings before drawing strong conclusions about sex differences and morbid curiosity. In addition, since the present studies involved samples with relatively young and highly educated participants, more research is needed to establish whether other demographic characteristics (e.g., age) may influence choice for negative information.

**References**

Bradley, M.M., Codispoti, M., Sabatinelli, D. & Lang, P.J. (2001). Emotion and motivation II: Sex differences in picture processing. *Emotion, 1,* 300-319.

Hamann, S. & Canli, T. (2004). Individual differences in emotion processing. *Current Opinion in Neurobiology, 14*, 233-238.

Kring, A.M., & Gordon, A.H. (1998). Sex differences in emotion: expression, experience and physiology. *Journal of Personality and Social Psychology,74*, 686-703.

Zaleski, Z. (1984). Sensation-seeking and preference for emotional visual stimuli. *Personality*

*and Individual Differences, 5,* 609-611.
