# Supplementary material for: Choosing the negative: A behavioral demonstration of morbid curiosity
Source: PLoS One. 2017 Jul 6;12(7):e0178399. doi: 10.1371/journal.pone.0178399 (PMC5500011; doi:10.1371/journal.pone.0178399)
Supplement: S1 Study — (DOCX) [file pone.0178399.s005.docx]

**Supporting Information Study 1**

*Choice between negative images.* When choosing between two negative images, participants preferred to view negative social images (M = .64; SD = .24) over negative physical images, *t*(49) = 4.18, *p* < .001, *d* = .59, and negative social images (M = .66; SD = .29) over negative nature images, *t*(49) = 4.02, *p* < .001, *d* = .55. Negative physical and negative nature images were chosen equally often, *t*(49) = -.54, *p* = .60.

*Subjective ratings of negativity, intensity, interest and complexity.* For the mean subjective rating of negativity, intensity and interest per image condition, please see Table 1 in this Supporting Information file.

Ratings of interest differed between categories, *F*(3,144) = 70.43, *p* < .001, η²_p_= .60. Follow-up paired samples *t*-tests demonstrated that all categories differed significantly from each other (*p’s* < .001). Negativity ratings differed between categories, *F*(3,144) = 440.30, *p* < .001, η²_p_= .90. Follow-up paired samples *t*-tests demonstrated that negative images from all categories were judged as more negative than neutral images (*p’s* < .001). Negative social images and negative physical images were more negative as negative nature images (*p’s* < .001). In line with the selection criteria used, negative social images and negative physical images did not differ in terms of negativity (*p* = .54).

Second, judgments of intensity differed between categories, *F*(3,144) = 340.51, *p* < .001, η²_p_= .88. Follow-up paired samples *t*-tests demonstrated that all categories differed significantly from each other (*p’s* < .001). Third, judgments of complexity differed between categories, *F*(3,144) = 136.83, *p* < .001, η²_p_= .74. Simple effects demonstrated that all negative categories were judged as more complex than neutral images (*p’s* < .001). Negative social images and negative physical images were more complex as negative nature images (*p’s* < .001). Negative social images and negative physical images only differed in complexity when testing against an uncorrected threshold (*p* = .038).

**Table 1. Overview subjective ratings.**

|  |  | Interest | Negativity | Intensity | Complexity |
| --- | --- | --- | --- | --- | --- |
| Study 1 | Negative social | 55.88 | 77.44 | 69.04 | 54.53 |
|  | Negative physical | 46.47 | 78.05 | 76.09 | 50.99 |
|  | Negative nature | 32.47 | 43.59 | 41.15 | 26.59 |
|  | Neutral | 21.43 | 9.11 | 9.9 | 20.1 |

Note: Table reflects mean ratings of interest, negativity, intensity and complexity (range 0 -100) for the different image conditions within Study 1.
