# Supplementary material for: Choosing the negative: A behavioral demonstration of morbid curiosity
Source: PLoS One. 2017 Jul 6;12(7):e0178399. doi: 10.1371/journal.pone.0178399 (PMC5500011; doi:10.1371/journal.pone.0178399)
Supplement: S2 Study — (DOCX) [file pone.0178399.s006.docx]

**Supporting Information Study 2**

*Spatial coherence and feature energy as indicators of visual complexity.* To verify that the images used in Study 2 were relatively well matched in terms of low-level visual features, two summary statistics (i.e., *feature energy* and *spatial coherence*) were calculated and compared across image conditions. Feature energy (FE) reflects the contrast that is present in an image and spatial coherence (SC) reflects to what extent the scene in an image is fragmented. Previous research has indicated that FE and SC are related to behavioral and neural indicators of image categorization (i.e., decisions about whether a scene is natural or artificial; Groen, Ghebreab, Prins, Lamme & Scholte, 2013).

Analyses of FE demonstrated some differences between the stimuli in terms of contrast strength. Negative and neutral physical images differed significantly in FE (*M* = .0048 vs. *M* = .0034; *p* = .008); as did negative and positive physical images (*M* = .0046 vs. *M* = .0034; *p* = .012) and negative and neutral nature images (*M* = .0052 vs. *M* = .0030; *p* = .001). Negative and neutral social images (*M* = .0057 vs. *M* = .0052; *p* = .315), negative and positive social images (*M* = .0052 vs. *M* = .0051; *p* = .943) and negative and positive nature images (*M* = .0042 vs. *M* = .0042; *p* = .931) did not differ in FE.

Analyses of SC demonstrated that the stimuli were similar in terms of scene fragmentation. Negative and neutral social images (*M* = 1.31 vs. *M* = 1.42; *p* = .114) and negative and positive social images (*M* = 1.26 vs. *M* = 1.33; *p* = .497) did not differ significantly in SC; nor did negative and neutral physical images (*M* = 1.26 vs. *M* = 1.13; *p* = .233); negative and neutral nature images (*M* = 1.27 vs. *M* = 1.14; *p* = .236) and negative and positive nature images (*M* = 1.23 vs. *M* = 1.30; *p* = .561). Only negative and positive physical images differed marginally in terms of SC (*M* = 1.35 vs. *M* = 1.10; *p* = .060).

This analysis of feature energy and spatial coherence demonstrated that the negative social stimuli used in Study 2 did not differ significantly in terms of low-level visual features as compared to the neutral and positive social alternatives. The physical and nature images did differ from their matched alternatives, but only in feature energy. Since previous research has indicated that spatial coherence, and not feature energy, is associated with decision-making processes (Groen et al., 2013), the fact that the negative images and their alternatives were well-matched in terms of spatial coherence is most relevant to the present choice paradigm.

*Subjective ratings of interest and complexity.* For the mean subjective rating of negativity, intensity and interest per image condition, please see Table 1 in this Supporting Information file.

Interest ratings differed between categories, *F*(2,98) = 5.24, *p* = .015 (with Greenhouse-Geisser correction), η²_p_= .10. Overall, participants rated social images (*M* = 42.99; *SE* = 1.68) as significantly (*p* < .001) more interesting than physical images (*M* = 37.56; *SE* = 1.50). Also nature images (*M* = 41.63; *SE* = 2.36) were rated as significantly more interesting than physical images (*p* < .05, uncorrected). Interest ratings also differed between valence conditions, *F*(2,98) = 44.19, *p* < .001, η²_p_= .47. Participants rated negative images (*M* = 51.77; *SE* = 2.23) as significantly (*p* < .001) more interesting than positive (*M* = 39.56; *SE* = 1.95) and neutral images (*M* = 30.85; *SE* = 1.95). Positive images were also rated as more interesting than neutral images (*p* < .001). Furthermore, there was an interaction between valence and category, *F*(4,196) = 57.08, *p* < .001, η²_p_= .54. Follow-up paired samples *t*-tests demonstrated that negative social images were rated as more interesting than all other image categories (*p’s* < .001, *d*_z_*’s* > .66). Negative physical images were rated as significantly more interesting than the remaining image categories (*p’s* < .001), except compared to positive nature images (*p* = .096, uncorrected).

Complexity ratings differed between categories, *F*(2,98) = 41.22, *p* < .001, η²_p_= .46. Overall, participants rated social images (*M* = 44.24; *SE* = 1.68) as significantly (*p* < .001) more complex than physical images (*M* = 35.26; *SE* = 1.43) and nature images (*M* = 30.68; *SE* = 2.51). Physical images were rated as significantly more complex than nature images (*p* < .01,). Complexity ratings also differed between valence conditions, *F*(2,98) = 154.93, *p* < .001, η²_p_= .76. Participants rated negative images (*M* = 54.92; *SE* = 1.67) significantly (*p* < .001) more complex than positive (*M* = 26.88; *SE* = 2.29) and neutral images (*M* = 28.37; *SE* = 2.01). Positive and neutral images did not differ in complexity (*p* = .19). Furthermore, there was an interaction between valence and category, *F*(4,196) = 86.65, *p* < .001, η²_p_= .64. The most relevant paired samples *t*-tests demonstrated that negative social and physical images were rated as more complex than all other image categories (*p’s* < .001), although they did not differ in complexity from each other (*p* = .93).

**Table 1. Overview subjective ratings.**

|  |  | Interest | Complexity |
| --- | --- | --- | --- |
| Study 2 | Negative social | 63.03 | 64.22 |
|  | Positive social | 37.38 | 33.41 |
|  | Neutral social | 28.57 | 35.09 |
|  | Negative physical | 54.27 | 64.13 |
|  | Positive physical | 33.65 | 21.20 |
|  | Neutral physical | 24.74 | 20.44 |
|  | Negative nature | 38.00 | 36.41 |
|  | Positive nature | 47.64 | 26.04 |
|  | Neutral nature | 39.23 | 29.60 |

Note: Table reflects mean ratings of interest and complexity (range 0 -100) for the different image conditions within Study 2.

*Correlations between subjective ratings and choice.* Replicating Study 1, choice for negative social images in the negative social – neutral social condition correlated with interest for negative social images, *ρ* = .34, *p* = .017. Choice for negative social images in the negative social – positive social condition also correlated with interest, *ρ* = .37, *p* = .009. Furthermore, again replicating Study 1, choice for negative physical images in the negative physical – neutral physical condition correlated with interest for negative physical images, *ρ* = .44, *p* = .001. Choice for negative physical images in the negative physical – positive physical condition also correlated with interest, *ρ* = .44, *p* = .001. Finally, again replicating Study 1, choice for negative nature images in the negative nature – neutral nature condition correlated with interest for negative nature images, *ρ* = .47, *p* = .001. Choice for negative nature images in the negative nature – positive nature condition also correlated with interest, *ρ* = .52, *p* < .001. Consistent with the findings from Study 1, there were no significant (negative) correlations between the choice scores and interest for neutral images or positive images, nor were there any correlations with the complexity ratings.

**References**

Groen, I.I.A., Ghebreab, S., Prins, H., Lamme, V.A.F, & Scholte, H.S.(2013). From image statistics to scene gist: evoked neural activity reveals transition from low-level natural image structure to scene category. *The Journal of Neuroscience, 33*, 18814-18824.
