# Supplementary material for: Choosing the negative: A behavioral demonstration of morbid curiosity
Source: PLoS One. 2017 Jul 6;12(7):e0178399. doi: 10.1371/journal.pone.0178399 (PMC5500011; doi:10.1371/journal.pone.0178399)
Supplement: S3 Study — (DOCX) [file pone.0178399.s007.docx]

**Supporting Information Study 3**

*Subjective ratings.* For the mean subjective rating of negativity, intensity and interest per image condition, please see Table 1 in this Supporting Information file.

Interest ratings differed between categories, *F*(1,54) = 34.80, *p* < .001, η²_p_= .39; social images (*M* = 38.01; *SE* = 1.55) were rated as more interesting than physical images (*M* = 31.23; *SE* = 1.61). Interest ratings also differed between valence conditions, *F*(2,108) = 163.95, *p* < .001, η²_p_= .75. Participants rated negative images (*M* = 54.36; *SE* = 2.00) as significantly (*p* < .001) more interesting than positive (*M* = 29.51; *SE* = 1.95) and neutral images (*M* = 19.99; *SE* = 1.61). Positive images were also rated as more interesting than neutral images (*p* < .001). An interaction between valence and category, *F*(2,108) = 32.23, *p* < .001, η²_p_= .37, indicated that negative social images were rated as more interesting than all other image categories (*p’s* < .001). Negative physical images were rated as significantly more interesting than positive and neutral physical images (*p’s* < .001).

Negativity ratings differed between valence conditions, *F*(2,108) = 1911.66, *p* < .001, η²_p_= .97. Negative images (*M* = 79.71; *SE* = 1.24) were rated as significantly (*p* < .001) more negative than positive images (*M* = 5.08; *SE* = .94) and neutral images (*M* = 7.73; *SE* = 1.24). Negativity ratings differed marginally between categories, *F*(1,54) = 3.07, *p* = .085, η²_p_= .054. Finally, there was a significant interaction between valence and category, *F*(2,108) = 7.48, *p* < .01, η²_p_= .12. The most relevant follow-up paired samples *t*-test demonstrated that, unlike in Study 1, negative physical images were rated as more negative than negative social images (*p* < .01).

Intensity ratings also differed between valence conditions, *F*(2,108) = 726.63, *p* < .001, η²_p_= .93; negative images (*M* = 74.03; *SE* = 1.34) were rated as significantly (*p* < .001) more intense than positive images (*M* = 21.28; *SE* = 2.21) and neutral images (*M* = 11.51; *SE* = 1.47). Intensity ratings also differed between categories, *F*(1,54) = 27.16, *p* < .001, η²_p_= .34; physical images (*M* = 37.57; *SE* = 1.39) were rated as more intense than social images (*M* = 33.64; *SE* = 1.47). Finally, there was a significant interaction between valence and category, *F*(2,108) = 17.72, *p* < .001, η²_p_= .25. The most relevant follow-up paired samples *t*-test demonstrated that intensity ratings differed between negative physical images and negative social images (*p* < .01).

**Table 1. Overview subjective ratings.**

|  |  | Interest | Negativity | Intensity |
| --- | --- | --- | --- | --- |
| Study 3 | Negative social | 62.94 | 77.95 | 69.19 |
|  | Positive social | 29.63 | 4.78 | 20.53 |
|  | Neutral social | 21.47 | 8.43 | 11.20 |
|  | Negative physical | 45.77 | 81.47 | 78.88 |
|  | Positive physical | 29.39 | 5.38 | 22.02 |
|  | Neutral physical | 18.52 | 7.03 | 11.82 |

Note: Table reflects mean ratings of interest, negativity and intensity (range 0 -100) for the different image conditions within Study 3.

*Correlations between subjective ratings and choice.* Replicating Study 1 and 2, choice for negative social images in the negative social – neutral social condition correlated with interest for negative social images, *ρ* = .32, *p* = .019. Choice for negative social images in the negative social – positive social condition did not correlate significantly with interest, *ρ* = .08, *p* = .581. Furthermore, again replicating Study 1 and 2, choice for negative physical images in the negative physical – neutral physical condition correlated with interest for negative physical images, *ρ* = .57, *p* < .001. Choice for negative physical images in the negative physical – positive physical condition also correlated with interest, *ρ* = .53, *p* < .001. Consistent with previous findings, there were no significant (negative) correlations between the choice scores and interest ratings for neutral or positive images, nor were there any correlations with the negativity and intensity ratings.
